# Thermoelectric La-doped SrTiO_3_ epitaxial layers with single-crystal quality: from nano to micrometers

**DOI:** 10.1080/14686996.2017.1336055

**Published:** 2017-06-20

**Authors:** Mihai Apreutesei, Régis Debord, Mohamed Bouras, Philippe Regreny, Claude Botella, Aziz Benamrouche, Adrian Carretero-Genevrier, Jaume Gazquez, Geneviève Grenet, Stéphane Pailhès, Guillaume Saint-Girons, Romain Bachelet

**Affiliations:** ^a^ Institut des Nanotechnologies de Lyon (INL) – CNRS UMR 5270, Ecully, France; ^b^ Institut Lumière Matière (ILM) - CNRS UMR 5306, Villeurbanne, France; ^c^ Institut d’Electronique et des Systèmes (IES) - CNRS UMR 5214, Univ. Montpellier 2, Montpellier, France; ^d^ Institut de Ciencia de Materials de Barcelona (ICMAB -CSIC), Bellaterra, Spain

**Keywords:** Functional oxides, Thermoelectricity, Molecular beam epitaxy, La-doped SrTiO_3_, Integrated films, 50 Energy materials, 302 Crystallization / Heat treatment / Crystal growth, 306 Thin film / Coatings, 210 Thermoelectronics / Thermal transport / insulators, 201 Electronics / Semiconductor / TCOs

## Abstract

High-quality thermoelectric La_0.2_Sr_0.8_TiO_3_ (LSTO) films, with thicknesses ranging from 20 nm to 0.7 μm, have been epitaxially grown on SrTiO_3_(001) substrates by enhanced solid-source oxide molecular-beam epitaxy. All films are atomically flat (with rms roughness < 0.2 nm), with low mosaicity (<0.1°), and present very low electrical resistivity (<5 × 10^−4^ Ω cm at room temperature), one order of magnitude lower than standard commercial Nb-doped SrTiO_3_ single-crystalline substrate. The conservation of transport properties within this thickness range has been confirmed by thermoelectric measurements where Seebeck coefficients of approximately –60 μV/K have been recorded for all films. These LSTO films can be integrated on Si for non-volatile memory structures or opto-microelectronic devices, functioning as transparent conductors or thermoelectric elements.

## Graphical abstract

**Figure:**
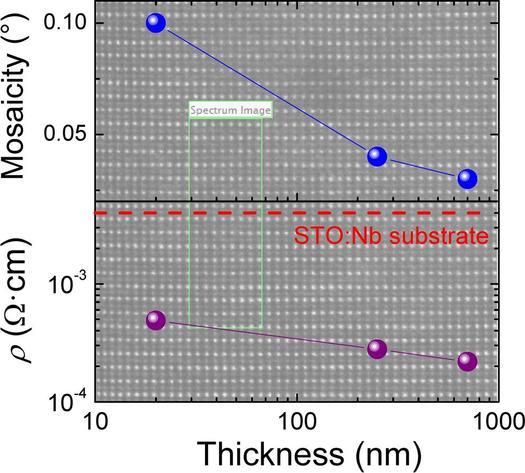

## 1. Introduction

Functional oxides present high chemical and thermal stability with a large degree of compositional freedom, which offers great flexibility to tune their physical properties [1,2]. Therefore, functional oxides containing non-toxic, abundant and cheap elements are alternative and viable candidates for many devices in large-scale application fields such as non-volatile memories, sensors, actuators or energy harvesters. In particular, perovskite oxides, of general formula ABO_3_, exhibit a wide range of properties such as ferroelectricity, piezoelectricity, pyroelectricity, ferromagnetism, thermoelectricity and metal-insulator transitions [1,2]. One of the best-known perovskite oxides is SrTiO_3_ (STO), a dielectric material at room temperature, which displays, for instance, tunable ferroelectric transition by isovalent Ba^2+^ substitution on A-site [3] and tunable metal-insulator (MI) transition by substituting La^3+^ on A-site, Nb^5+^ on B-site or by introducing oxygen vacancies [4]. In this light, specific La^3+^ doping level in SrTiO_3_, La_x_Sr_1-x_TiO_3_ (LSTO), can result in high values of electrical conductivity (up to ~10^4^ S/cm at 300 K), Seebeck coefficient (up to ~1 mV/K at 300 K), thermoelectric power factor (up to ~40 μW cm^−1^ K^−2^ at 300 K, larger than those of Bi_2_Te_3_ family materials) [4,5], electron mobility (up to ~10^4^ cm^2^ V^−1^ s^−1^ at low temperatures) [6] and optical transparency in the visible range [7]. LSTO could therefore be used in important applications as transparent conductor [7], thermoelectric element [5] or non-volatile memory involving MI transitions or field effects [8–10]. Nevertheless, its physical properties strongly depend on several parameters during the elaboration process that impacts the atomic and chemical structure. Since applications require the use of high-quality films, epitaxial growth of single-crystalline films of controlled properties over a wide thickness range is a prerequisite. However, the results published so far on epitaxial LSTO films are limited to films of a few nanometers thickness and contain incomplete experimental or structural details.

Nanometric epitaxial LSTO films have been grown mainly using pulsed laser deposition (PLD) [7,11,12] and a particular hybrid molecular beam epitaxy (MBE) technique involving an organo-metallic precursor as Ti source [5,6]. Since oxygen vacancies act as an additional *n*-type dopant [7], the transport properties of the La-doped STO films can be altered by varying the oxygen partial pressure P(O_2_) during deposition [5,7,11]. The LSTO grown by PLD can present low resistivity (*ρ*) of the order of 10^−3^ Ω cm. For instance, *ρ* = 4.6 × 10^−3^ Ω cm has been reported with 15% La doping in films grown at P(O_2_) = 10^−3^ Torr [7]. Decreasing P(O_2_) below 10^−5^ Torr results in a resistivity drop by a factor of ~2 due to the introduction of oxygen vacancies, in agreement with the increased carrier density [11,12]. Electrical resistivity of nanometric LSTO films grown by MBE is about one order of magnitude lower than that of films grown by PLD, taking into account the same La doping level and the same substrate [7,13]. For instance, with 15% La doping, a resistivity down to few 10^−4^ Ω cm can be reached by MBE at room temperature even after consequent suppression of oxygen vacancies by air-annealing [13]. This difference can be due to (i) the non-equilibrium energetic character of PLD process, (ii) the higher residual background pressures of the PLD deposition chambers and (iii) the non-obvious stoichiometric transfer from the target to the film due to the dependence on laser fluence and difference in volatility or sticking coefficients between elements [14]. Non-stoichiometric transfer occurs with Pb-, Bi-, K- and Ru-based oxides [15,16], and even with La-based oxides; it is a possible reason for observing *n*-type conductivity at the LaAlO_3_/SrTiO_3_ interface [17]. Therefore, MBE appears as a reference equilibrium high-purity elaboration technique for the growth of state-of-the-art conducting oxide films with controlled composition. However, most of the MBE-grown LSTO films have a thickness t < 150 nm, or are prepared by a particular hybrid MBE method [5,6,13]. This is likely due to the main challenge of solid-source oxide MBE systems to ensure the stability and reproducibility of the fluxes and to control the stoichiometry. Here, epitaxial LSTO single-layers are grown by enhanced solid-source oxide MBE up to ~μm in thickness, maintaining excellent structural quality (low mosaicity down to 0.03°) and low electrical resistivity (< 5 × 10^−4^ Ω cm at room temperature). Additionally, growth conditions with the correlated structural and functional properties of the layers are described in detail.

## 2. Experimental details

Epitaxial La_0.2_Sr_0.8_TiO_3_ (LSTO) films were grown on (001)-oriented SrTiO_3_ (STO) substrates by solid-source oxide MBE using effusion cells, metallic elements and molecular oxygen. This elaboration technique allows the monolithic integration of perovskite films on Si via a SrTiO_3_ buffer-layer that assists the fabrication of oxide-based devices for the microelectronic industry [18,19]. The crucibles used in the effusion cells are made of tungsten for high-temperature refractory-Ti evaporation (promoting its challenging flux stability), pyrolytic boron nitride (PBN) for Sr and tantalum for La. Net fluxes were measured by a Bayard–Alpert ionization gauge with thoria-coated Ir filament located at the sample position in the center of the chamber, subtracting the residual background pressures at different temperatures (see Figure S1 in supplementary material). A low residual base pressure of about 10^−10^ Torr is achieved in the MBE chamber by using combined turbo-molecular and liquid nitrogen cryogenic pumping. Before growth, the substrates were annealed for 10 min under a di-oxygen partial pressure of 10^−7^ Torr, in the MBE chamber, at a temperature of 600 °C. The epitaxial growth has been performed in co-deposition process at a constant substrate temperature of 450 °C, using a di-oxygen partial pressure of 10^−7^ Torr (avoiding oxidation of the metallic sources and thus flux deviations) and a growth rate of ~1.5 monolayers/min (ML/min). Growth rate and film thickness were estimated by X-ray reflectometry (XRR) and cross-checked by transmission electron microscopy. Film thickness ranged from 20 nm to 0.7 μm. *In situ* reflection high-energy electron diffraction (RHEED) was employed to monitor the structural quality of the film during the growth. The LSTO films were further annealed *ex situ* at 450 °C in a tubular furnace under air during several hours in order to eliminate the oxygen vacancies in both films and substrates to ensure reliability of the transport properties measurements [20]. The substrates were then checked to be well insulating (Figure S2 in supplementary material). The film morphology was analyzed using atomic force microscopy (AFM) in tapping mode. Their crystalline quality was investigated using X-ray diffraction (XRD) (SmartLab diffractometer from Rigaku equipped with a high brilliance 9 kW X-ray source, two-bounce Ge(220) monochromator, and two crossed R_x_-R_y_ cradles enabling precise alignment). The stoichiometry of 20 nm LSTO films and chemical composition of the surface region of thicker films were determined by X-ray photoelectron spectroscopy (XPS) with Al Kα photon source energy at 1486.6 eV. In supplementary material, one representative example of the core level spectra is illustrated in Figure S3, and the methodology for the chemical composition extraction is given. One film’s microstructure was examined with a NION UltraSTEM scanning transmission electron microscope (STEM) operated at 100 kV and equipped with a NION aberration corrector and a Gatan Enfina spectrometer for electron energy loss spectroscopy (EELS). Electrical resistivity measurements were performed in Van der Pauw geometry from room to low (10 K) temperature using a 4-Watt ARS displex refrigeration system. For this purpose, ultrasonic wire bonding (West-Bond machine) was used to connect aluminum wires to Cu bonding pads on the corners of the squared samples. Carrier concentration and mobility were measured by Hall effect at room temperature. Seebeck coefficient measurements, using the differential method, S = ΔV/ΔT where ΔV is the thermo-emf induced by the thermal gradient ΔT (see Figure S3 in supplementary material), were performed from room temperature to ~100 K using a similar device as the one described by Bérardan et al. [21].

## 3. Results and discussion

**Figure 1.**
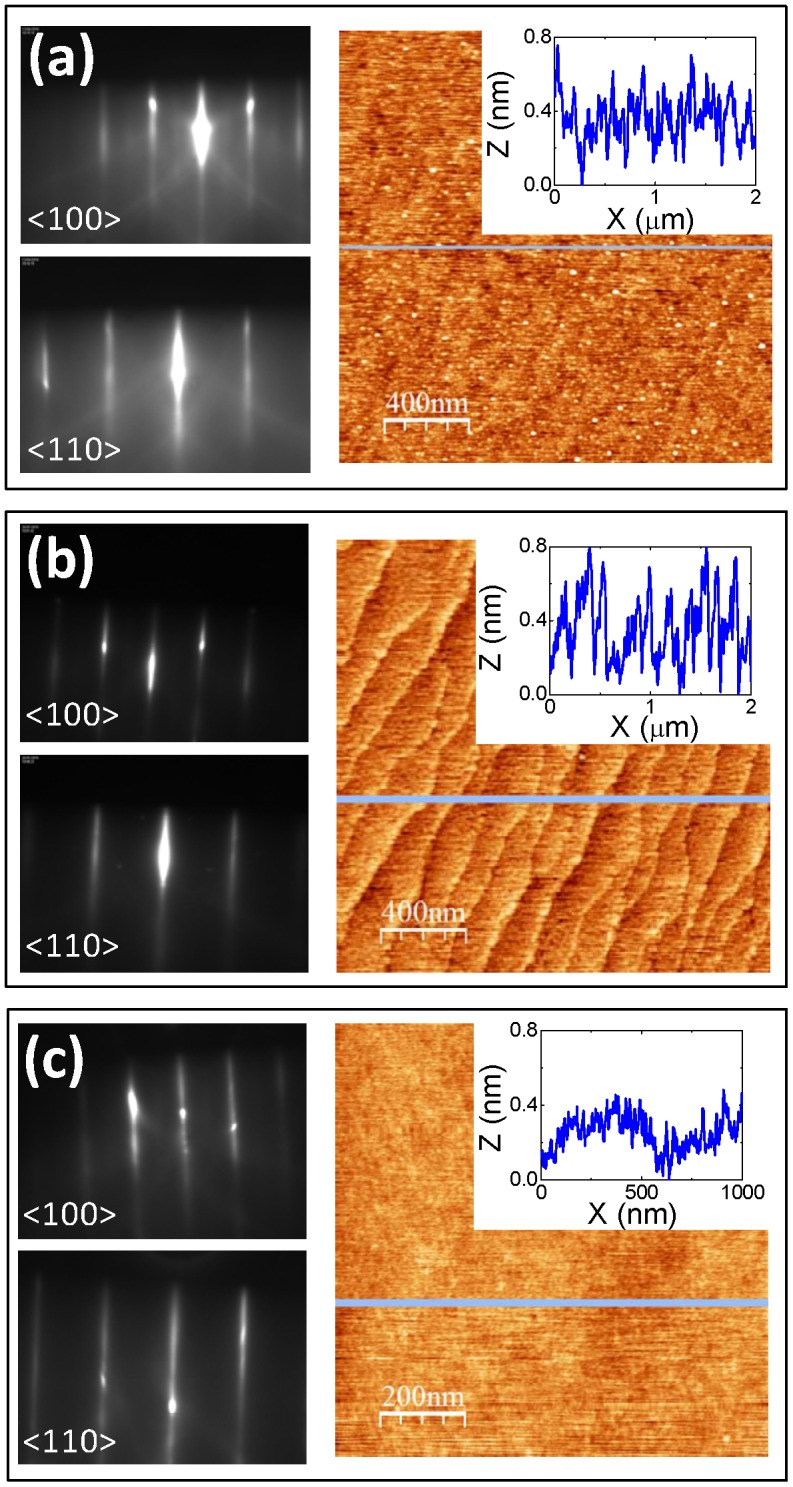
(left) RHEED patterns along the <100> and <110> directions after growth, and (right) AFM topographic images and corresponding topographic profiles of (a) 20 nm thick, (b) 250 nm thick and (c) 0.7 μm thick LSTO layers.

LSTO epitaxial films of different thicknesses, from 20 nm up to 0.7 μm, were grown on (001)-oriented STO substrates. The RHEED patterns of the as-grown LSTO films are shown in Figure 1 (left). For all studied thicknesses, RHEED patterns present high diffraction contrast with entire-order reflection streaks along the <110> and <100> directions after growth, indicating that the films are epitaxial with stoichiometric and atomically flat surface [22]. The flatness of the films is confirmed by the AFM topographic images (Figure 1, right), where root-mean-square (rms) roughness less than 0.2 nm and peak-to-peak height amplitude less than one unit-cell (~0.4 nm) are measured for all thicknesses. XPS results show that, despite a possible La/Sr deviation from the targeted doping at the surface region, the cationic stoichiometry A/(A+B) is respected within 1.5% deviation (Table 1).

**Table 1.** Summary of the structural, chemical, electrical and thermoelectric properties of the LSTO films at room temperature. Note that the chemical composition (La at% and Sr at%) is measured by XPS in the surface region of the films (depth analysis less than 10 nm) and has been normalized to Ti.

Film	*t*	[La]	[Sr]	[A/(A+B)]	*c*	Mosaicity	*ρ*	*S*	*n*	*μ*
	(nm)	(at%)	(at%)	(at%)	(Å)	(°)	(10^−4^ Ω cm)	(μV/K)	(10^21^ cm^−3^)	(cm^2^ V^−1^ s^−1^)
A	20	19.1	84.2	50.8	3.920	0.10	5.0	−74	2.7	5.5
B	250	27.9	77.8	51.4	3.925	0.04	3.0	−61	4.3	5.5
C	700	29.2	72.0	50.3	3.940	0.03	2.2	−51	5.5	4.7

**Figure 2.**
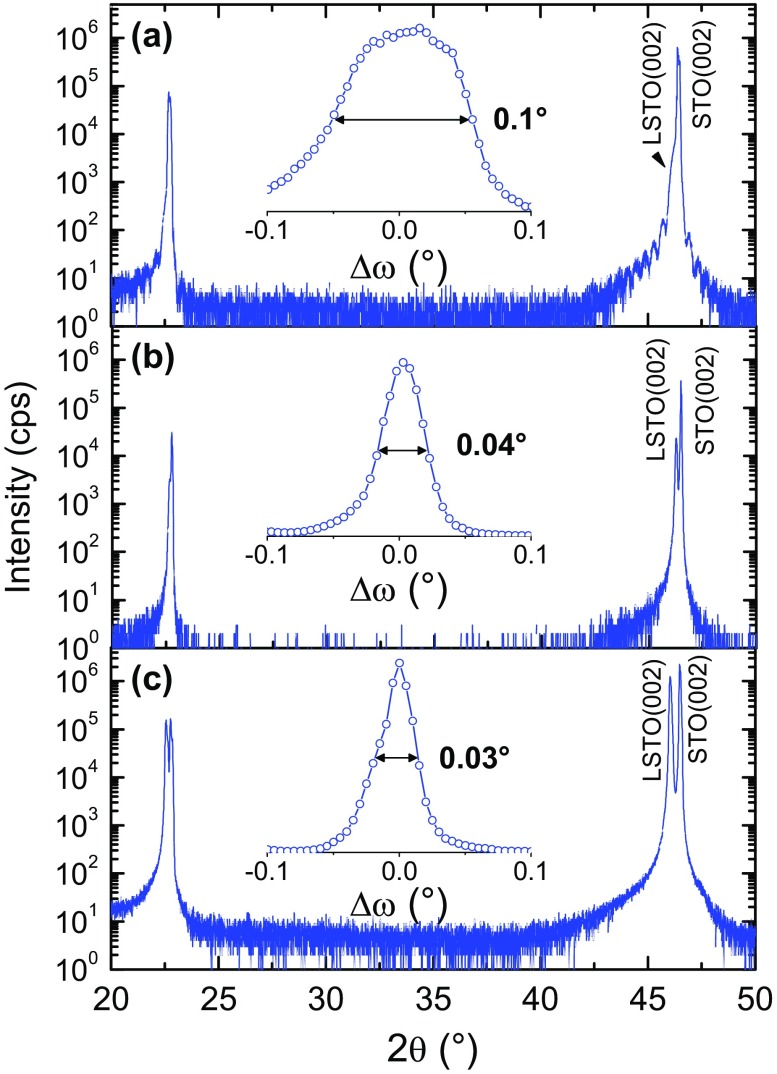
XRD θ-2θ symmetrical scans, and ω-scans around the (002) LSTO reflection in inset, of (a) 20 nm thick, (b) 250 nm thick and (c) 0.7 μm thick LSTO layers.

The structural properties of the LSTO films have been measured by XRD (Figure 2). Apart from the intense Bragg diffraction peaks corresponding to the {00l} reflections of the LSTO film and STO substrate, no other reflections were observed along the [00l] out-of-plane direction, showing that the films are clearly single-oriented. The LSTO (002) reflection of the 20 nm thick film shows Pendellösung fringes, indicating good crystalline quality with atomically flat interfaces (Figure 2(a)). As expected, the diffracted intensity increases with the thickness of the film, whereas the peak width decreases. The measured out-of-plane lattice parameters (*c*) were found to be in-between 3.92 and 3.94 Å. Although slightly larger than theoretical values [13,23], the measured lattice parameters are well in the range of those reported by other groups [11–13]. In particular, they are closer to theoretical values than those of PLD films grown under P(O_2_) lower than 10^−1^ Torr, for which the *c* values increase up to 4.08 Å [11]. The lattice parameter of the films depends on P(O_2_) during deposition, epitaxial strain, doping concentration and also cationic stoichiometry deviation [14]. Based on the work of Ohnishi et al., the lattice parameter expansion, that is less than 0.05 Å here, corresponds to a maximum of 3% cationic stoichiometry deviation, in agreement with our XPS results (Table 1) [14]. Importantly, a full width at half maximum (FWHM) of 0.1° of the (002) reflection rocking curve of the 20 nm thick film (inset of Figure 2(a)) demonstrates the low mosaicity of the film, which is better than reported by other groups [7,13]. Furthermore, the mosaicity value decreases when increasing the film thickness, reaching a lower value of 0.03° for 0.7 μm thick LSTO film (inset of Figure 2(c)), which is close to that of a STO single-crystalline substrate.

**Figure 3.**
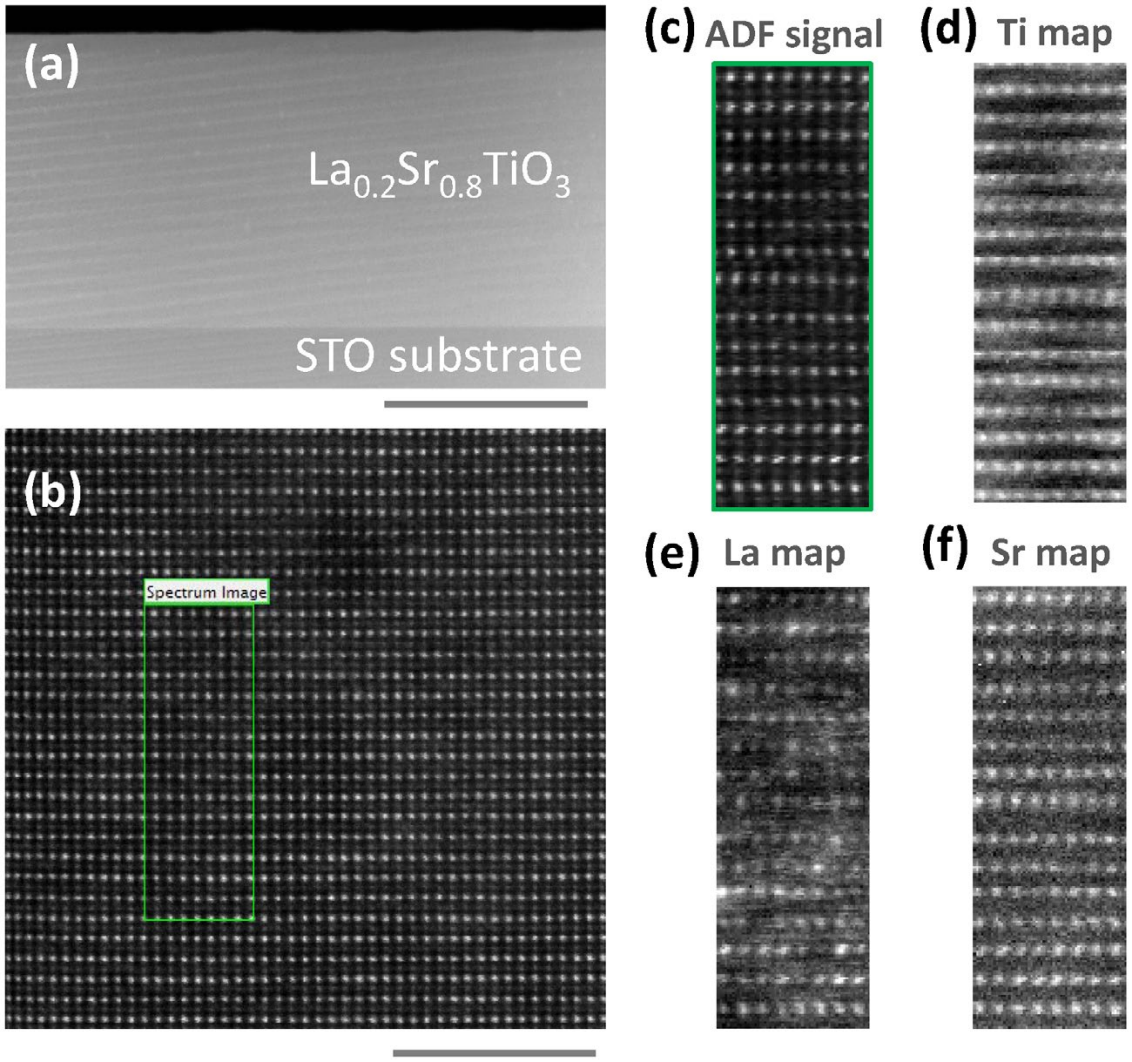
(a) Low-magnification and (b) high-magnification Z-contrast images of a 250 nm thick LSTO layer viewed along the STO[110] zone axis. Scale bars represent 200 nm in (a) and 4 nm in (b). (c) Annular-dark field (ADF) image of the region marked in (b). (d–f) EELS elemental maps of the region marked in (b) corresponding to Ti L_2,3_, La M_4,5_ and Sr L_2,3_ edges, respectively.

To illustrate the high structural quality of the LSTO films, a STEM characterization has been carried out on a 250 nm thick film (Figure 3). Figure 3(a) shows a low-magnification Z-contrast image which reveals a defect-free and continuous over a long lateral length LSTO layer. The stripes in the low magnification image are scanning moiré fringes, which appear when the pixel size of scanning grating is close to the lattice plane spacing. A higher-magnification Z-contrast image of the lattice shows a perfect LSTO crystallinity, with no structural distortions, neither in the La/Sr nor in the Ti sublattices (Figure 3(b)). Atomically resolved EELS spectrum images were also acquired to study any possible chemical ordering (Figure 3(c)–(f)). The elemental maps show that the ABO_3_ lattice is preserved, with the Sr substitution by La in the A-site of the perovskite structure. The introduction of La within the STO matrix does not form superstructures. Different EELS spectrum images acquired from different sample regions have shown that the La does not form clusters and that it is homogeneously distributed within the layer.

**Figure 4.**
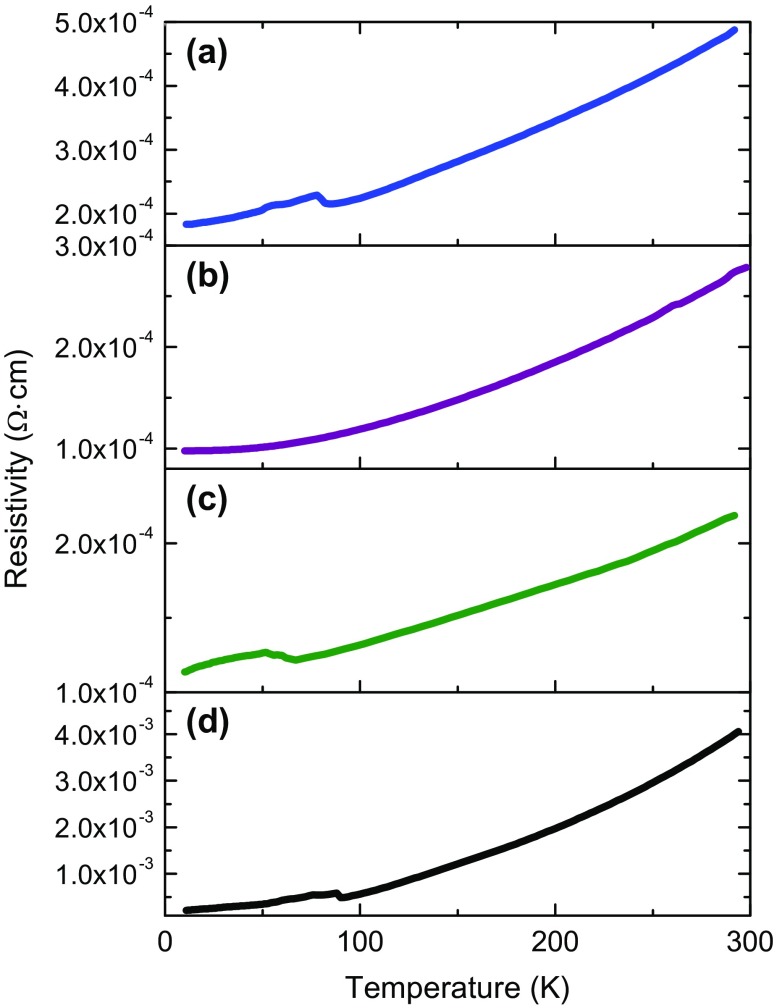
Temperature dependence of the electrical resistivity of (a) 20 nm thick, (b) 250 nm thick and (c) 0.7 μm thick LSTO layers and (d) single-crystalline 1.4 at% (0.7 wt%) Nb-doped STO(001) substrate.

Figure 4(a)–(c) shows the electrical resistivity of the LSTO films as a function of temperature for all the thickness range. A standard commercial (1.4 at%, so commonly referenced as 0.7 wt%) Nb-doped STO single-crystalline substrate has been measured for comparison (Figure 4(d)). All the LSTO films present low resistivity below 5 ×10^−4^ Ω cm at room temperature, about one order of magnitude lower than Nb-doped STO single-crystalline substrate (4 ×10^−3^ Ω cm, in agreement with the provider’s data sheets). The temperature dependence exhibits the characteristic T^2^ dependence of a Fermi liquid (see Figure S5 in Supplementary Material) as previously observed experimentally in doped STO over a wide temperature and doping ranges [5,7,24,25]. The resistivity values of our LSTO films with 20% targeted La doping are consistent with the reports of other groups, with respect to doping concentration and strain state [5,13]. The measured carrier concentration (from 2.7 to 5.5 × 10^21^ cm^−3^) and mobility at room temperature (in-between 4.7 and 5.5 cm^2^ V^−1^ s^−1^) are also consistent with the measured structural/chemical properties (Table 1) and literature data [5,7,11,13]. La surface segregation may occur during deposition or air-annealing at 450 °C, creating a La-rich LSTO surface in thick films. Yet XPS results are consistent with the thickness-averaged structural and electrical data, despite XPS being a surface-sensitive technique. Lattice parameters and carrier concentrations are higher in thicker films in which La concentration at the surface region is measured to be more than the expected 20%, that could come from minor flux deviations during the long depositions.

**Figure 5.**
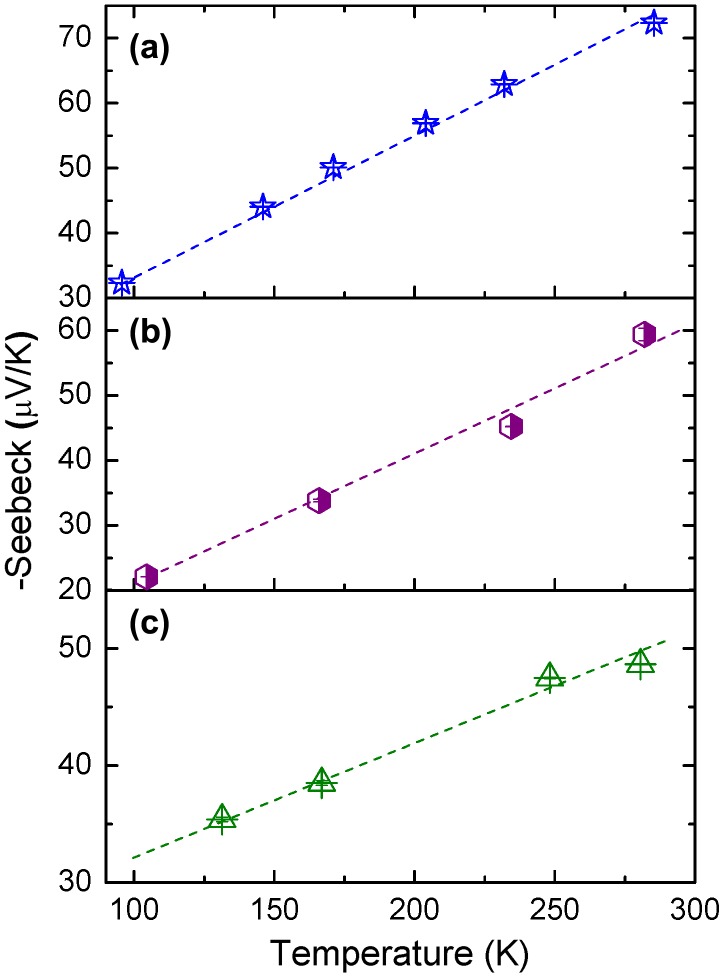
Temperature dependence of the Seebeck coefficient of (a) 20 nm thick, (b) 250 nm thick and (c) 0.7 μm thick LSTO layers.

Seebeck coefficient S was measured from room temperature down to 100–150 K (Figure 5). Its room-temperature value ranged between –51 and –74 μV/K for all films, in perfect agreement with the doping level, carrier concentration (Table 1) and previous reports [5]. Consistently, the minor measured S differences are inversely proportional to differences of resistivity values. It is worth noting that the thermoelectric properties of doped STO was recently very well reproduced within four orders of magnitude for electrical conductivity and Seebeck coefficient in the context of a theoretical tight-binding approach based on a fully realistic band Hamiltonian, where only the electron-electron scattering process is considered [26]. Further, more complex thermoelectric structures yielding enhanced thermopower, such as superlattices with fractional monolayer control for instance [27], can fully take advantage of the MBE elaboration tool (flexibility of the doping concentration controlled at the monolayer level).

## 4. Conclusions

Highly conductive thermoelectric LSTO epitaxial films with excellent structural quality have been grown by solid-source oxide MBE up to ~μm thickness range. All the films have atomically flat surface and low mosaicity (< 0.1°) and present low electrical resistivity (< 5 × 10^−4^ Ω cm), one order of magnitude lower than standard commercial Nb-doped STO single-crystals. Seebeck coefficient measurements have confirmed the electrical properties. STEM images have shown the high structural quality and homogeneity of the film. The minor variations of the transport properties are consistent with the slight variation of doping concentration and carrier density. These results underline the potential of La-doped STO films grown by standard MBE technique for applications as transparent conductor or thermoelectric element for which specific doping concentration or μm thick integrated layers are required.

## Disclosure statement

No potential conflict of interest was reported by the authors.

## Funding

This work was supported by Horizon 2020 Framework Programme [grant number Project TIPS / ICT-02-2014-1-644453].

## Supplemental data

The supplemental material for this paper is available online at https://doi.org/10.1080/14686996.2017.1336055.

## Supplementary Material

2017-05-24_Suppl_Mat.docx
