# Supplementary material for: Thermoelectric La-doped SrTiO3 epitaxial layers with single-crystal quality: from nano to micrometers
Source: Sci Technol Adv Mater. 2017 Jun 20;18(1):430–5. doi: 10.1080/14686996.2017.1336055 (PMC5507149; doi:10.1080/14686996.2017.1336055)
Supplement: 2017-05-24_Suppl_Mat.docx [file tsta_a_1336055_sm9283.docx]

Thermoelectric La-doped SrTiO_3_ epitaxial layers with single-crystal quality: from nano to micrometers

Mihai Apreutesei^a^, Régis Debord^b^, Mohamed Bouras^a^, Philippe Regreny^a^, Claude Botella^a^, Aziz Benamrouche^a^, Adrian Carretero-Genevrier^a,c^, Jaume Gazquez^d^, Geneviève Grenet^a^, Stéphane Pailhès^b^, Guillaume Saint-Girons^a^ and Romain Bachelet^a*^

^a^ Institut des Nanotechnologies de Lyon (INL) – CNRS UMR 5270, Ecole Centrale de Lyon, Bâtiment F7, 36 av. Guy de Collongue, 69134 Ecully Cedex, France. *E-mail: romain.bachelet@ec-lyon.fr

^b^ Institut Lumière Matière (ILM) - CNRS UMR 5306, UCBL, Bât. Léon Brillouin, Campus LyonTech La Doua, 69622 Villeurbanne Cedex, France.

^c^ Institut d’Electronique et des Systèmes (IES), CNRS, Universite Montpellier 2, 860 rue de Saint Priest, 34095 Montpellier, France.

^d^ Institut de Ciencia de Materials de Barcelona (ICMAB -CSIC) Campus UAB, E-08193 Bellaterra, Catalunya, Spain.

^*^ E-mail: romain.bachelet@ec-lyon.fr

**Content of the Supplementary Material:**

1. MBE net fluxes measured by Bayard-Alpert gauge for each metallic element (Fig. S1)

2. Photographs of LSTO epitaxial film before and after air-annealing (Fig. S2)

3. XPS spectra of the La4d, Sr3d, Ti2p and O1s core levels (Fig. S3) and methodology used to extract the chemical composition

4. Seebeck coefficients measurements around room temperature (Fig. S4)

5. T^2^-dependence of the resistivity (Fig. S5)

**1. MBE net fluxes measured by Bayard-Alpert gauge**

**Figure S1:** MBE net fluxes as a function of temperature of (a) Ti, (b) Sr, and (c) La effusion cells. (d) MBE net fluxes for La and Sr, as well as the expected carrier concentration (n_3D_), as a function of the La doping concentration (x) in La_x_Sr_1-x_TiO_3_ (LSTO). The star points in (a-c) represent the net fluxes and corresponding effusion cell temperatures used in this paper for x=0.2.

Figure S1 shows the net flux for each metallic element (measured using the Bayard-Alpert gauge under UHV and after subtraction of the background pressure) at different effusion cells temperatures within targeted composition range (La_0.2_Sr_0.8_TiO_3_). The logarithm of the net flux increases linearly with temperature, as expected [S1-S2]. Net flux as small as a few 10^-10^ Torr (in the order of magnitude of the residual base pressure) can be finely measured with the procedure detailed above. The net flux of Ti is set to 4 x 10^-9^ Torr, corresponding to a cell temperature of around 1500 °C and to a growth rate of 1.5 ML/min (Fig. S1a). The corresponding 20% La doping and consequent 80% Sr, to maintain a cationic stoichiometry ratio A/B = 1, is obtained at a La net flux of 2 × 10^-9^ Torr for a temperature around 1310°C, and a Sr net flux of 1.1 × 10^-8^ Torr for a temperature of ~440°C, respectively (Fig. S1(b-d)). The overall chemical composition of 20 nm thick LSTO films were estimated using XPS measurements where relative elemental concentrations were obtained, crosschecked with surface reconstructions by *in-situ* RHEED [S3]. Based on these results, the working temperatures of each effusion cell were adjusted accordingly to obtain the desired composition.

**2. Photographs of LSTO epitaxial film**

**Figure S2:** (a-b) Photographs of 20 nm thick epitaxial LSTO film on SrTiO_3_(001) substrate (a) before and (b) after air-annealing at 450°C of a few hours. (c) Photograph of an as-received SrTiO_3_(001) substrate, for comparison.

Figure S2 shows that the oxygen vacancies created during MBE growth under low oxygen partial pressure (yielding grey colour) can be suppressed by air annealing at 450°C for several hours after deposition. Then, the backsides of the substrates have been checked to be well electrically insulating.

**3. XPS spectra and methodology to extract the chemical composition**

**Figure S3:** Representative XPS spectra of (a) La 4d, (b) Sr 3d, and (c) Ti 2p, and (d) O 1s core levels, taken here on the 0.7 μm thick LSTO film after air annealing at 450°C.

Figure S3(a-d) displays typical XPS spectra of La 4d (a), Sr 3d (b), Ti 2p (c) and O 1s (d) core levels, taken here on the thickest LSTO film after air annealing. The peak positions correspond to well oxidized LSTO. The low value of full-width at half-maximum (FWHM) of the O 1s core level peak (1.3 eV) attests the good crystalline quality of the LSTO layer. Besides, the carrier concentration was estimated based on the XPS results and confirmed by Hall effect measurements on 20 nm thick films. It was found that the La donors are ionized and each La donates one electron to the conduction band of SrTiO_3_ as expected in this doping range [S4].

The relative chemical composition is extracted using the following relation of the total spectral intensity of each element (in films thicker than 10 nm):

I = σ λ N_at_ ,

where σ is the photoionization cross-section of the considered element and core-level [S5], λ is the mean free path in the material [S6], and N_at_ is the number of considered atoms. Knowing σ and λ, and after Shirley background subtraction of each spectra, the chemical composition of the analyzed region (less than 10 nm in SrTiO_3_) can be extracted with a precision of around 5%.

**4. Seebeck coefficients measurements**

**Figure S4:** Seebeck coefficients (S = ΔV/ΔT) measured around room temperature of (a) 20 nm, (b) 250 nm, and (c) 0.7 μm thick LSTO films.

**5. T^2^-dependence of the resistivity**

**Figure S5:** Resistivity of the 250 nm thick LSTO film plotted in function of T^2^.

Figure S5 shows that the T^2^ dependence of the resistivity of the LSTO films is verified in a wide temperature range above ~40 K.

**References** (of the supplementary Material)

[S1] Henini M, editor. Molecular Beam Epitaxy - from research to mass production. [place unknown (USA)]:Elsevier. 2013.

[S2] Demkov A, Posadas AB. Integration of functional oxides with semiconductors. New York (NY): Springer. 2014.

[S3] Kajdos AP, Stemmer S. Surface reconstructions in molecular beam epitaxy of SrTiO_3_. Appl Phys Lett. 2014;105:191901.

[S4] Son J, Moetakef P, Jalan B, et al. Epitaxial SrTiO_3_ films with electron mobilities exceeding 30,000 cm^2^ V^-1^ s^-1^. Nat Mater. 2010;9:482.

[S5] https://vuo.elettra.eu/services/elements/WebElements.html

[S6] use for instance the NIST "electron inelastic mean-free-path" database (imfpwin)
